# Methods for the isolation and analysis of Aβ from postmortem brain

**DOI:** 10.3389/fnins.2023.1108715

**Published:** 2023-01-26

**Authors:** Wei Hong, Wen Liu, Alexandra O. Desousa, Tracy Young-Pearse, Dominic M. Walsh

**Affiliations:** ^1^Laboratory for Neurodegenerative Research, Ann Romney Center for Neurologic Diseases, Brigham and Women’s Hospital and Harvard Medical School, Boston, MA, United States; ^2^The Brain Cognition and Brain Disease Institute, Shenzhen Institute of Advanced Technology, Chinese Academy of Sciences, Shenzhen, Guangdong, China; ^3^Ann Romney Center for Neurologic Diseases, Brigham and Women’s Hospital and Harvard Medical School, Boston, MA, United States

**Keywords:** amyloid β-protein, soluble aggregates, amyloid plaques, induced neurons, neurotoxicity

## Abstract

Amyloid β-protein (Aβ) plays an initiating role in Alzheimer’s disease (AD), but only a small number of groups have studied Aβ extracted from human brain. Most prior studies have utilized synthetic Aβ peptides, but the relevance of these test tube experiments to the conditions that prevail in AD is uncertain. Here, we describe three distinct methods for studying Aβ from cortical tissue. Each method allows the analysis of different ranges of species thus enabling the examination of different questions. The first method allows the study of readily diffusible Aβ with a relatively high specific activity. The second enables the analysis of readily solubilized forms of Aβ the majority of which are inactive. The third details the isolation of true Aβ dimers which have disease-related activity. We also describe a bioassay to study the effects of Aβ on the neuritic integrity of iPSC-derived human neurons. The combined use of this bioassay and the described extraction procedures provides a platform to investigate the activity of different forms and mixtures of Aβ species, and offers a tractable system to identify strategies to mitigate Aβ mediated neurotoxicity.

## 1. Introduction

Compelling evidences from genetic, neuropathological, biochemical and biomarker studies have shown that the amyloid β-protein (Aβ) plays a central role in Alzheimer’s disease (AD) (Jack et al., 2010; Selkoe and Hardy, 2016; McDade and Bateman, 2017). Recent clinical data suggest that certain monoclonal antibodies (e.g., lecanemab, aducanumab, and donanemab) which facilitate the removal of amyloid plaques can slow cognitive decline in mild AD patients (Sevigny et al., 2017; Mintun et al., 2021; Swanson et al., 2021a,b; Budd Haeberlein et al., 2022; van Dyck et al., 2022). Human Aβ can exist in myriad forms differing in primary structure, conformation and size, and the identity of the most disease-relevant species remains unknown (Mc Donald et al., 2015; Wildburger et al., 2017; Lau et al., 2021).

Our prior work has shown that the primary structure of Aβ from human brain is highly heterogeneous with a diversity of N- and C-termini (Mc Donald et al., 2015; Brinkmalm et al., 2019). In contrast, most studies assessing Aβ aggregation, structure and activity have utilized synthetic peptide of a single sequence (Yankner and Lu, 2009). Only a few groups have studied Aβ extracted from human brain. These studies have been limited to those investigating the structure and composition of Aβ in amyloid plaques (Allsop et al., 1983; Glenner and Wong, 1984; Masters et al., 1985; Roher et al., 1996; Welander et al., 2009), or so-called soluble Aβ (Suzuki et al., 1994; Kuo et al., 1996, 1997; Roher et al., 1996; Shinkai et al., 1997; Enya et al., 1999; Mc Donald et al., 2015; Brody et al., 2017; Mukherjee et al., 2021). The latter are defined operationally and refer to any form of Aβ released by homogenization in aqueous buffer and which remain in solution following high-speed centrifugation.

Here, we describe three protocols for the preparation of Aβ-containing AD brain extracts and a paradigm to investigate AD relevant bioactivity. The first section of the protocol details two methods to extract aqueous soluble forms of Aβ. Method I involves soaking thin slices of brain in artificial cerebrospinal fluid (aCSF). Method II utilizes a more traditional homogenization procedure. Method III describes a protocol that enables isolation of size exclusion separated pure Aβ dimers and monomers. Finally, we detail the use of human iPSC-derived glutamatergic neurons and live-neuron imaging to measure the neurite disrupting activity of various Aβ-containing extracts.

## 2. Materials and equipment

Here we provide details of vendors and catalog numbers for all chemicals and reagents we used for the experiments described. However, common reagents/chemicals from other sources should work equally well.

### 2.1. Reagents

• Milli-Q water.
• NaCl (Sigma-Aldrich, cat. no. S7653).
• KCl (Sigma-Aldrich, cat. no. P9333).
• NaH_2_PO_4_ (Sigma-Aldrich, cat. no. S8282).
• NaHCO_3_ (Sigma-Aldrich, cat. no. S5761).
• Leupeptin (Sigma-Aldrich, cat. no. L2884).
• Aprotinin (Sigma-Aldrich, cat. no. A1153).
• Pepstatin A (Sigma-Aldrich, cat. no. P5318).
• EGTA (Sigma-Aldrich, cat. no. E4378).
• Pefabloc (Sigma-Aldrich, cat. no. 76307).
• NaF (Sigma-Aldrich, cat. no. S7920).
• 0.5 M EDTA (Invitrogen, cat. no. 15575-020).
• Protein A sepharose (Sigma-Aldrich, cat. no. P3391).
• Anti-Aβ polyclonal antibody S97 (D. Walsh lab).
• Pre-immune serum (D. Walsh lab).
• Trizma-base (Sigma-Aldrich, cat. no. T6066).
• Hydrochloric Acid (Fisher Scientific, cat. no. A144).
• BSA (Sigma-Aldrich, cat. no. A2153).
• Sodium tetraborate (Sigma-Aldrich, cat. no. S9640).
• Dimethyl pimelimidate (DMP) (Sigma-Aldrich, cat. no. D8388).
• Ethanolamine (Sigma-Aldrich, cat. no. E-9508).
• Glycine (Sigma-Aldrich, cat. no. G7126).
• NaN_3_ (Sigma-Aldrich, cat. no. 71289).
• 20% Sodium dodecyl sulfate (Millipore, cat. no. 428018).
• β-mercaptoethanol (Sigma-Aldrich, cat. no. 6250).
! CAUTION This reagent is highly caustic. Do not allow its contact with the skin or eyes. Use safety goggles and an 8-inch face shield when handling.
• Sucrose (Sigma-Aldrich, cat. no. 84097).
• Congo red (Sigma-Aldrich, cat. no. C6277).
• 88% formic acid (Fisher Scientific, cat. no. A118P-100).
! CAUTION This reagent is highly caustic. Do not allow its contact with the skin or eyes. Use safety goggles and an 8-inch face shield when handling.
• Ammonium bicarbonate (Sigma-Aldrich, cat. no. 09830).
• Blue dextran (Sigma-Aldrich, cat. no. D4772-1VL).
• Gel filtration standard (Bio-Rad, cat. no. 1511901).
• mTeSR Matrix (Corning, cat. no. 354230).
• mTeSR medium-1L kit (Stemcell Tech, cat. no. 85857).
• Penicillin/Streptomycin (Invitrogen, cat. no. 15070063).
• Y-27632 (Stemcell Tech, cat. no. 72304).
• Accutase (Stemcell Tech, cat. no. 07920).
• PBS (Invitrogen, cat. no. 14190250).
• DMSO (Sigma-Aldrich, cat. no. D2650).
• Neurobasal™ Medium (Invitrogen, cat. no. 21103-049).
• DMEM/F12 (Invitrogen, cat. no. 11330057).
• GlutaMAX™ Supplement (Invitrogen, cat. no. 35050).
• Puromycin (Invitrogen, cat. no. A11138-03).
• Doxycycline Hyclate (Sigma-Aldrich, cat. no. D9891).
• Dextrose (Sigma-Aldrich, cat. no. D9434).
• N2 supplement B (Stemcell Tech, cat. no. 07156).
• MEM non-essential amino acids (Gibco, cat. No. 1140-050).
• KnockOut™ DMEM (Gibco, cat. No. 10829018).
• Knockout Replacement Serum (Gibco, cat. No. 10828−028).
• Blocker A (Meso Scale Discovery, cat. no. R93BA).
• 4xMSD Read Buffer P (Meso Scale Discovery, cat. no. R92PC).
• Streptavidin SULFO-TAG Labeled (Meso Scale Discovery, cat. no. R32AD).

### 2.2. Equipment

• Tissue chopper (McIlwain, model no. TC572).
• Bench top centrifuge (Eppendorf, model no. 5810R).
• Ultracentrifuge (Beckman, model no. Optima L-90K).
• Overhead stirrer (Wheaton, model no. 903475).
• 15 mL tissue grinder (Wheaton, model no. 358010).
• Lyophilizer (Labconco, model no. FreeZone).
• FPLC system (GE HealthCare).
• Light microscope with a polarizing filter (Leica).
• Water bath and dry bath.
• Lab vacuum system.
• IncuCyte live-cell video microscopy system (Essen Bioscience).
• Double-Edge Stainless blade (VWR, cat. no. 100491-888).
• Scalpel blade (Aspen Surgical Products, cat. no. 372622).
• Superdex 75 10/300 column (GE HealthCare, cat. no. 17-5174-01).
• HiTrap desalting column (GE HealthCare, cat. no. 17-1408-01).
• Zeba spin 40k MWCO desalting column (Fisher Scientific, cat. no. 87773).
• Slide-A-Lyzer G2 dialysis cassettes, 2000 MWCO (Fisher Scientific, cat. no. 87721).
• Cover slips (Fisher Scientific, cat. no. S17525B).
• 96-well micro plate (Greiner, cat. no. 655096).
• 0.2 μm Syringe filters (VWR, cat. no. 28145-501).
• 0.2 μm Membrane filters (Sigma-Aldrich, cat. no. 58060-U).
• 1 mL Syringes (BD, cat. no. 309628).
• 10 mL Syringes (BD, cat. no. 309604).
• 50 mL falcon tubes (Fisher Scientific, cat. no. 14-432-22).
• 13.2 and 38.5 mL Ultracentrifuge tubes (Beckman, cat. no. 344059; cat. no. 344058).
• 1.5 mL protein low bind tubes (Eppendorf, cat. no. 13-698-794).
• 2 mL protein low bind tubes (Eppendorf, cat. no. 13-698-795).
• 1.5 mL protein low bind tubes (Eppendorf, cat. no. 13-698-794).
• 40 μm Sterile Cell Strainers (Corning, cat. no. 431750).
• 112 μm nylon mesh filter (cat. no. U-CMN-112-A).
• 38 μm nylon mesh filter (cat. no. U-CMN-38-A).
• 30 mL Syringe (BD, cat. no. 305618).

### 2.3. Recipes for solutions used in protocol

#### 2.3.1. 10 × aCSF based buffer (aCSF-B, pH 7.4)

Dissolve 72.5 g NaCl, 2.1 g KCl, 1.5 g NaH_2_PO_4_, and 21.8 g NaHCO_3_ in 800 mL distilled water. Bring to 1 L with Milli-Q water and filter sterilize. Store at 4°C.

#### 2.3.2. aCSF extraction buffer: aCSF based buffer containing protease inhibitors

Add 10 mL of 10 × aCSF-B to 80 mL distilled water. Add 1 mL of 0.5 mg/ml leupeptin, 1 mL of 0.5 mg/ml aprotinin, 1 mL of 0.2 mg/ml pepstatin, 1 mL of 12 mg/ml pefabloc, 1 mL of 0.1 M EGTA, 1 mL of 0.5 M EDTA, and 1 mL of 0.5 M sodium fluoride. Bring to 100 mL with Milli-Q water and filter sterilize. Store at 4°C.

#### 2.3.3. Resuspension buffer for protein A sepharose beads: 50 mM Tris-HCl, pH 7.6 containing 150 mM NaCl and 2% BSA

Dissolve 3.03 g Trizma-base in 300 mL Milli-Q water and adjust pH to 7.6. Add 4.38 g NaCl and 10 g BSA. When dissolved bring to 500 mL with Milli-Q water and filter sterilize.

#### 2.3.4. Antibody coupling buffer: 0.2 M sodium borate, pH 9.0

Dissolve 38.14 g sodium borate in 300 mL Milli-Q water and heat to dissolve. Adjust pH to 9.0 with HCl. Bring to 500 mL with Milli-Q water and filter sterilize.

#### 2.3.5. Stop buffer for antibody coupling: 0.2 M ethanolamine

Add 6.035 mL Ethanolamine (density 1.012 g/mL, stock concentration 16.568 M) to 500 mL with Milli-Q water. Adjust pH to 8.0 with approximately 18 mL of 5N HCl and filter sterilize.

#### 2.3.6. Wash buffer A for antibody coupling: 100 mM glycine, pH 3.0

Dissolve 3.755 g of glycine in 400 mL Milli-Q water. Adjust pH to 3.0 with HCl. Bring to 500 mL with Milli-Q water and filter sterilize.

#### 2.3.7. Wash buffer B for antibody coupling: 100 mM Tris-HCl, pH 8.0

Dissolve 12.11 g Tris-base in 400 mL Milli-Q water. Adjust pH to 8.0 with HCl. Bring to 500 mL with Milli-Q water and filter sterilize.

#### 2.3.8. Plaque extraction buffer: 50 mM Tris-HCl containing 2% SDS and 0.1 M β-mercaptoethanol, pH 7.6

Dissolve 3.03 g Tris-base in 300 mL Milli-Q water and adjust pH to 7.6. Add 50 mL of 20% SDS and 3.5 mL β-mercaptoethanol. When dissolved bring to 500 mL with Milli-Q water and filter sterilize.

#### 2.3.9. 1% Congo red staining solution

Dissolve 1 g Congo red in 90 mL Milli-Q water, bring to 100 mL and filter sterilize.

#### 2.3.10. Wash buffer for plaque extraction: 150 mM NaCl, 0.1% SDS

Dissolve 4.38 g NaCl in 300 mL Milli-Q water, add 2.5 mL 20% SDS. When dissolved bring to 500 mL with Milli-Q water and filter sterilize.

#### 2.3.11. Sucrose gradient solutions for plaque isolation: 1.2–1.8 M sucrose in 50 mM Tris-HCl, pH 7.6 containing 1% SDS

Dissolve 4.84 g Tris-base in 200 mL Milli-Q water and adjust pH to 7.6, add 40 mL of 20% SDS. Mix thoroughly and split into 4, 50 mL lots placing each in a separate bottle. Add 82.15 g (1.2 M), 95.84 g (1.4 M), 109.54 g (1.6 M), and 123.23 g (1.8 M) sucrose into the four bottles, respectively. When dissolved, bring each solution to 200 mL with Milli-Q water.

#### 2.3.12. Blue dextran solution

Dissolve 50 mg blue dextran in 25 mL of Milli-Q water and filter sterilize.

#### 2.3.13. Mobile phase of FPLC: 50 mM ammonium bicarbonate, pH 8.5

Dissolve 3.953 g ammonium bicarbonate in 900 mL of Milli-Q water. Adjust pH to 8.5 with ammonium hydroxide. Bring to 1 L with Milli-Q water and filter sterilize.

#### 2.3.14. BDNF stock solution (1000×): 10 μg/ml in PBS containing 0.1% BSA

Dissolve one vial of 10 μg BDNF in 1 mL of PBS containing with 0.1% BSA. Mix thoroughly and aliquot into 100 μL vials. Store at −20°C.

#### 2.3.15. CNTF stock solution (1000×): 10 μg/ml in PBS containing 0.1% BSA

Dissolve one vial of 10 μg CNTF in 1 mL of PBS containing with 0.1% BSA. Mix thoroughly and aliquot into 100 μL vials. Store at −20°C.

#### 2.3.16. GDNF stock solution (1000×): 10 μg/ml in PBS containing 0.1% BSA

Dissolve one vial of 10 μg GDNF in 1 mL of PBS containing with 0.1% BSA. Mix thoroughly and aliquot into 100 μL vials. Store at −20°C.

#### 2.3.17. Y-compound (Y-27632): 10 mM in DMSO

Dissolve one vial of 1 mg Y-27632 compound in 312.2 μL of DMSO. Filter sterilize and aliquot into 50 μL vials. Store at −20°C.

#### 2.3.18. Doxycycline hyclate stock solution: 20 mg/ml in distilled water

Dissolve 1 g doxycycline hyclate (DOX) in 50 mL Milli-Q water. Filter sterilize and aliquot into 100 μL vials. Store at −20°C.

#### 2.3.19. 20% dextrose stock solution

Dissolve 20 g of D-(+)-Glucose in 100 mL of Milli-Q water. Filter sterilize and store at 4°C.

#### 2.3.20. mTeSR medium

Add 100 mL of mTeSR™1 5× Supplement and 5 mL of Penicillin/Streptomycin (5,000 U/ml) to 400 mL of mTeSR™1 Basal Medium. Filter sterilize and store at 4°C.

#### 2.3.21. iN induction medium A: KSR medium containing 2 μg/mL doxycycline

Add 75 mL of KnockOut Serum Replacement, 5 mL of MEM non-essential amino acids, 5 mL of GlutaMAX™ Supplement and 0.5 mL of β-mercaptoethanol to 414.5 mL of KnockOut™ DMEM. Filter sterilize, store at 4°C, and shield from light. This medium is designated as KSR base medium. Prior to use, take 10 mL of the KSR base medium and add 1 μL of DOX stock solution.

#### 2.3.22. iN induction medium B: KSR:N2B medium containing 10 μg/mL puromycin and 2 μg/mL doxycycline

Add 5 mL of GlutaMAX™ Supplement, 7.5 mL of 20% Dextrose stock solution and 5 mL of N2-supplement B to 482.5 mL of DMEM/F-12 Medium. Filter sterilize and store at 4°C. This medium is designated as N2B base medium. Prior to use, add 5 mL of the KSR base medium to 5 mL of the N2B base medium, and then add 10 μL of Puromycin and 1 μL of DOX stock solution.

#### 2.3.23. iN induction medium C: N2B medium containing 1% B27, 10 μg/mL puromycin, and 2 μg/mL doxycycline

Prior to use, add 100 μL of B27 supplement, 10 μL of Puromycin and 1 μL of DOX stock solution to 10 mL of N2B base medium.

#### 2.3.24. Supplemental neurobasal medium

Add 5 mL of GlutaMAX™ Supplement, 7.5 mL of 20% Dextrose stock solution and 2.5 mL MEM non-essential amino acids to 485 mL of Neurobasal™ Medium. Filter sterilize and store at 4°C.

#### 2.3.25. iN cell culture medium

For 50 mL iN cell culture medium, add 1 mL of B27 supplement, 50 μL of BDNF, 50 μL of CNTF, 50 μL of GDNF, 50 μL of Puromycin, 5 μL of Doxycycline to 49 mL of Supplemental Neurobasal medium. Y-27632 is only required when plating iN cells, but not for iN maintenance.

#### 2.3.26. Medium to buffer-exchange brain extracts for application to neurons

For 100 mL buffer exchange medium, add 2 mL of B27 supplement and 1 mL of GlutaMAX™ Supplement to 97 mL of Neurobasal™ Medium. Filter sterilize and store at 4°C. Note: since buffer-exchanged extracts will be applied to iN cultures all manipulations are done under sterile conditions.

## 3. Stepwise procedure

### 3.1. Isolation of soluble Aβ from human brain tissue

#### 3.1.1. Brain tissue dissection (estimated time: 1 h)

Extreme care should be taken when handling human tissue. Experimenters should wear appropriate personal protective equipment and consumable materials should be disinfected in bleach prior to disposable in hazardous waste. Where possible experimenters should work in an isolated area, and the area should be thoroughly clean at the end of each session.

1) Before starting the procedure, set up tissue chopper (thickness = 0.5 mm, force = 50%) and pre-cool the cutting board on ice.
2) Remove brain tissue from −80°C storage and thaw slightly on dry ice. Cut the brain tissue into half gram chunks using a razor blade. Carefully remove blood vessels and white matter, keep gray matter.
3) Install pre-cooled cutting board on tissue chopper and chop brain tissue into ø 0.5 mm chunks. Divide chopped tissue in two: one half is used for Method 1 and the other for Method 2.
  Note: We here describe an example procedure appropriate to 20 g of dissected brain tissue. But we recommend adjusting experimental scales optionally for particular purposes. Brain tissue were obtained from four patients who both died with AD at Braak stage VI and temporal cortex used in the current study.

#### 3.1.2. Extraction methods (estimated time: 3 h)

##### 3.1.2.1. Method I

1) Prepare two, 5 g lots of chopped brain tissue and transfer each into two separate 50 mL ice-cold falcon tubes. Add 25 mL (20% w/v) of ice-cold extraction buffer (i.e., aCSF-B containing with protease inhibitors) to each tube and incubate with gentle side-to-side shaking at 4°C for 30 min.
  Note: Avoid harsh shaking. Thirty minutes incubation enables soluble proteins, including Aβ, to diffuse out of brain tissue.
2) Centrifuge the suspension at 2,000 × *g* and 4°C for 10 min, collect the upper 90% supernatant and transfer to clean ice-cold ultracentrifuge tubes.
3) Centrifuge at 200,000 × *g* and 4°C for 110 min using a SW41 Ti rotor. Carefully collect the upper 90% supernatant. The resulted sample is designated S extract.
  Note: Low speed centrifugation described in step 2 prior to ultracentrifugation is used to minimize physical disruption of plaques.

##### 3.1.2.2. Method II

1) Add 50 mL (20% w/v) of ice-cold aCSF-B (pH 7.4) with protease inhibitors to 10 g of chopped brain tissue and homogenize with 25 strokes of a Teflon-glass Dounce homogenizer.
2) Centrifuge at 200,000 × *g* for 110 min and 4°C in a SW41 Ti rotor. Carefully remove the upper 80% supernatant. The resulted sample is designated H extract.

#### 3.1.3. Dialysis to remove low molecular weight compounds from brain extracts (estimated time: 3 days)

1) During ultracentrifugation, prepare pre-cooled dialysis buffer (i.e., 1 × aCSF-B, pH 7.4) with a volume of at least 100-fold excess of designated brain extracts.
2) Add 50 mL of pre-cooled dialysis buffer to a new dialysis cassette and leave at 4°C for at least 10 min.
  Note: Ensure the cassette is cold prior to adding brain extract and there is no buffer leaking.
3) Remove dialysis buffer in the dialysis cassette and then add brain extract using a 10 mL pipette. Place cassette in the 5 L bucket with pre-cooled dialysis buffer and stir slowly for 24 h.
4) Replace dialysis buffer. Repeat the above step 3 twice.
  Note: We recommend dialysis against >100-fold excess of aCSF-B at 4°C, with three buffer changes over a 72 h period. The efficiency can be estimated by measuring the amount of glutamate in the final dialyzate vs. the starting extract (Figure 2). Typically, >90% of glutamate is removed by this regimen. Glutamate concentration was measured using the Amplex^®^ Red Glutamic Acid/Glutamate Oxidase Assay Kit (Invitrogen, cat. no. A12221) following manufacturer’s instructions.

**FIGURE 1:**
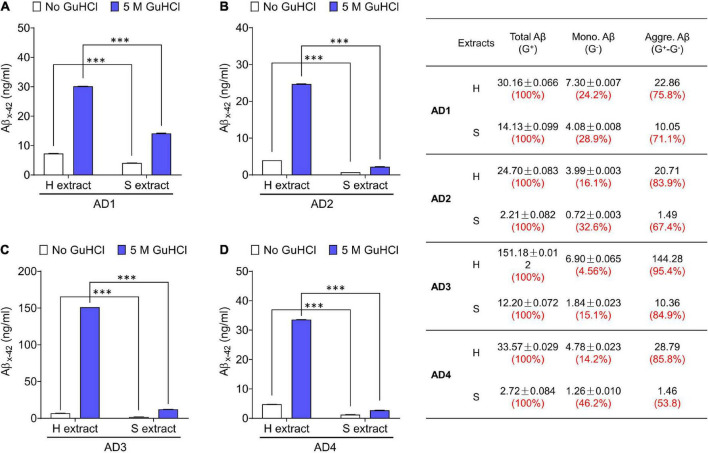
Aqueous extracts of AD brains contain a mixture of Aβ monomers and soluble aggregates. **(A–D)** The MSD-immunoassay preferentially detects monomeric Aβx-42. H extracts and S extracts from four AD brains were assessed for Aβ content with and without pre-incubation in 5 M GuHCl. Aβ measured without incubation in GuHCl is attributed to Aβ monomer, whereas Aβ detected in the homogenate pre-treated with GuHCl is the product of Aβ aggregates dissociated to monomer, plus native monomer. The table indicates the relative amount of Aβ present as native monomer and Aβ in aggregates dissociable with 5 M GuHCl. G– and G+ denote no GuHCl and 5 M GuHCl, respectively. Values were shown as mean ± SD. Treatments were examined by one-way ANOVA and significant differences are denoted as ^***^p < 0.001.

**FIGURE 2:**
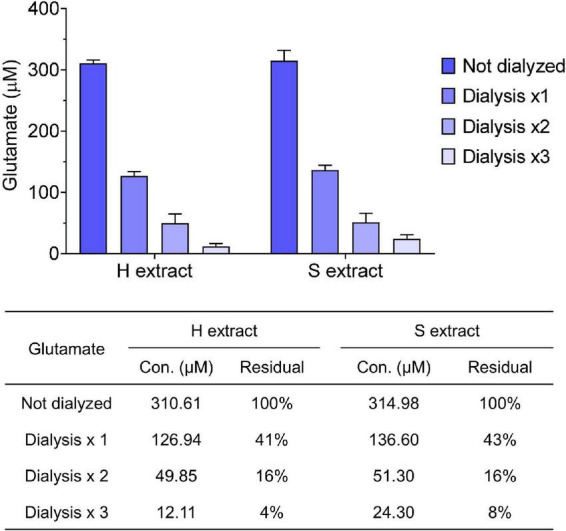
Small molecules are effectively removed from samples by dialysis. Glutamate concentration in human brain extracts was measured prior to and after each round of dialysis. The table shows the average values measured and the calculated residual percentage. Values were shown as mean ± SD.

5) Remove sample from the cassette using a 5 mL pipette and aliquot into 1.5 mL protein-low bind tubes. Freeze on dry ice and store at −80°C.
  Note: As Aβ are aggregation prone and sensitive to temperature changes, brain extracts can be thawed once and used. In general, remove samples from −80°C and allow to thaw at room temperature for 20 min.

#### 3.1.4. Conjugation of antibody to protein A beads (estimated time: 3 days)

Antibodies are used to deplete brain extracts of Aβ to provide a negative control for bioactivity studies. Polyclonal antibodies are covalently cross-linked to Protein A sepharose (PAS) beads to minimize the potentially confounding effects of free antibody.

1) Dissolve 0.25 g of PAS in 10 mL of 50 mM Tris-HCl, pH 7.6 containing 150 mM NaCl and 2% BSA. Incubate the PAS suspension with gentle side-to-side shaking at room temperature for 30 min.
2) During incubation, use a Zeba spin desalting column to buffer exchange 3∼4 mg of protein A purified anti-Aβ polyclonal antibody (e.g., S97 in our study) or control antibody [e.g., pre-immune serum (PIS)] into 50 mM Tris-HCl, pH 7.6 containing 150 mM NaCl.
  Note: Pre-immune serum is a negative control to anti-Aβ polyclonal antibody and does not recognized Aβ. If polyclonal antibody such as S97 is not available, a commercial antibody 4G8 is optional (Yang et al., 2017).
3) Centrifuge PAS suspension at 3,000 rpm for 5 min at room temperature and carefully discard supernatant.
4) Keep the PAS pellet (∼1 mL from 0.25 g dried PAS) and add 3 mg anti-Aβ polyclonal antibody. Leave stand for 1 h at room temperature and then incubate with gentle side-to-side shaking at room temperature for 1 h.
5) Centrifuge at 3,000 rpm for 5 min at room temperature and discard the supernatant. Wash PAS pellet with 10 mL of antibody coupling buffer (0.2 M sodium borate, pH 9.0). Centrifuge and discard supernatant. Repeat one time.
6) Resuspend PAS with 10 mL of antibody coupling buffer and add 50 mg of DMP, then incubate with gentle side-to-side shaking at room temperature for 30 min.
  Note: This coupling reaction requires pH > 8.3, adjust with NaOH if necessary.
7) Centrifuge at 3,000 rpm for 5 min at room temperature and discard supernatant. Resuspend PAS pellet with 10 mL of 0.2 M ethanolamine.
8) Centrifuge at 3,000 rpm for 5 min at room temperature and discard supernatant. Resuspend PAS pellet with 10 mL of 0.2 M ethanolamine and incubate with gentle side-to-side shaking at room temperature for 2 h.
9) Centrifuge at 3,000 rpm for 5 min at room temperature and discard supernatant. Resuspend PAS pellet with 1.75 mL PBS and transfer the suspension into two 2 mL Eppendorf tubes.
10) Centrifuge at 3,000 rpm for 5 min at room temperature and discard supernatant. Resuspend PAS pellet with 1.75 mL of 100 mM glycine for a minimal time interval.
11) Centrifuge at 10,000 rpm for 30 s at room temperature and discard supernatant. Resuspend PAS pellet with 1.75 mL of 100 mM Tris-HCl, pH 8.0.
12) Centrifuge at 3,000 rpm for 5 min at room temperature and discard supernatant. Resuspend each tube of 0.5 mL PAS pellet with 0.5 mL PBS containing 0.02% NaN_3_, yielding a 50% S97-PAS or PIS-PAS slurry.
  Note: Covalently conjugated antibodies can be stored at 4°C for up to 3 months and maintain high immunoreactivity.

#### 3.1.5. Aβ immunodepletion (estimated time: 3 days)

1) Thaw appropriate aliquots of brain extracts on ice. Add 30 uL S97-PAS [immunodepletion (ID)] or PIS-PAS [mock immunodepletion (mock-ID)] to each 0.5 mL brain extracts, gently mix by hand and then rotate at 4°C overnight.
2) Centrifuge at 6,000 rpm for 5 min at 4°C, carefully collect supernatant using gel loading tip and transfer into new 1.5 mL pro-low bind tubes. Keep the pellet as IP’d material.
3) Repeat the above step 2 twice. The final supernatant is designated as Aβ-ID’d or mock-ID’d brain extract.
4) Analyze the IP’d material from each round by Western Blot if necessary.
  Note: We recommend using a combination of anti-Aβ monoclonal antibodies specific for Aβ40 (e.g., 2G3) and Aβ42 (e.g., 21F12) to probe blots.
5) Measure Aβx-42 monomer concentration in Aβ-ID’d or mock-ID’d brain extracts using Aβx-42 immunoassay. Quantitate the efficiency of immunodepletion by comparing Aβ-ID’d and mock-ID’d brain extract. Perform assays using the Meso Scale Discovery (MSD) platform and reagents from Meso Scale (Rockville, MD, USA).
  Note: The Aβx-42 immunoassay preferentially detects Aβ monomers ending at Ala42. It employs m266 (3 μg/mL) for capture and biotinylated 21F12 (0.4 μg/mL) for detection (Mc Donald et al., 2015).
6) Measure aggregated Aβx-42 in Aβ-ID’d or mock-ID’d brain extracts by first dissociating aggregate in chaotrop. Incubate 20 μL of extract with 50 μL of 7 M GuHCl overnight at 4°C. Thereafter dilute samples 1:10 with assay diluent so that the final GuHCl concentration is 0.5 M. Quantitate the efficiency of immunodepletion by comparing Aβ-ID’d and mock-ID’d brain extract.
  Note: Previously we have shown that Aβ aggregates in AD brain extracts can be dissociated with chaotrops such as GuHCl, and that incubation of aggregate-containing extracts with GuHCl allows their disassembly and quantitation using monomer-preferring immunoassays. To match the buffer composition of standards with samples, prepare monomeric stock of Aβ1–42 in assay diluent containing 0.5 M GuHCl.

### 3.2. Isolation of amyloid plaques and purification of Aβ from human brain tissue (method III)

#### 3.2.1. Pre-screening of plaque-positive brain tissue (estimated time: 1 day)

Amyloid plaque isolation is time-consuming and the distribution and abundance of amyloid plaques in different brains and in different areas of the same brain is highly variable. Therefore, we recommend conducting a less time-consuming preliminary screen which involves randomly selecting a small piece of tissue from different areas, and comparing the abundance of Congo red stained plaques using light microscopy. Thus with this approach we identify regions of brain most likely to give good yields of plaque. Here we show an example of using temporal cortex from an end-stage AD patient.

1) Remove brain tissue from −80°C storage place on dry ice and allow to thaw slightly. Dissect frozen cortical tissue with a scalpel blade and selecting ∼100 mg aliquots of tissue from the different areas of the available tissue.
2) Mince tissue aliquots with a scalpel and transfer to 1 mL homogenizing tube.
3) Add 5 volumes of 2% SDS buffer and homogenize 20 strokes with a Teflon-glass Dounce homogenizer.
4) Transfer the homogenate to labeled 2 mL low-bind tubes. Heat at 100°C for 5 min and then cool down to room temperature.
5) Centrifuge at 10,000 rpm for 10 min at room temperature. Carefully remove supernatant using gel-loading tip attached to vacuum suction unit.
6) Vortex pellet in remaining buffer and remove 5 μL using wide-orifice tip and place onto a slide marked with Super Pap Pen.
7) Add 100 μL of 1% Congo red staining solution to each slide. Ensure the solution spread over sample.
8) Incubate slide at room temperature for 30 min and mount with a coverslip.
9) Visualize stained amyloid plaques by using a light microscope fitted with a polarizing filter. Count the number of birefringent cores. Based on this preliminary screen select the tissue that contain the largest number of Congo red positive plaques for plaque isolation.
  Note: Amyloid structures can be visualized using polarized light. Amyloid plaques have a characteristic Maltese cross appearance and undergo red/green birefringence when view under polarized light. This birefringence is most apparent when gently rotating the polarizing filter.

#### 3.2.2. Isolation of crude plaques from human brain tissue (estimated time: 1 day)

In general, a large amount of cortical tissue (i.e., 100 g of wet tissue) is required for the isolation of amyloid plaques. Here we describe an example procedure appropriate to 100 g of brain tissue.

1) Slightly thaw brain tissue on dry ice and dissect tissue away visible blood vessels and white matter. Mince 50 g of gray matter with a scalpel and then transfer to a 500 mL glass beaker.
2) Add 250 mL of plaque extraction buffer and incubate at room temperature for 2 h without shaking.
  Note: During the incubation period, set water bath to 100°C and the bench-top centrifuge to 26°C.
3) Homogenize the suspension with 20 strokes with a Teflon-glass Dounce homogenizer. Pool homogenates (approximately 300 mL from 50 g of dissected gray matter) from the same subject into a 500 mL glass beaker.
4) Place the homogenates in 100°C water bath for 15 min and then cool to room temperature.
5) Pass the homogenates through 112 um nylon mesh, transfer the flow through into six 50 mL tubes.
6) Centrifuge at 300 × *g* for 30 min at 26°C using a Bench top centrifuge.
7) Remove the supernatant leaving at least 15 mL liquid above the soft pellet.
  Note: Critical step. Avoid disrupting the soft pellet.
8) Combine the material from 6 tubes into 3 tubes. Rinse the emptied tubes three times with 5 mL 0.1% SDS solution and combine all materials together. Bring the content of each tube to 50 mL with 0.1% SDS solution.
  Note: At this stage, samples are quite sticky, thus we recommend rinsing emptied tubes thoroughly and retain all the materials.
9) Centrifuge at 300 × *g* for 30 min at 26°C using a Bench top centrifuge.
  (10) Remove the supernatant leaving at least 10 mL liquid above the pellet.
  Note: The pellet should be more compact than that in step 7, but still needs to avoid disruption.
11) Combine the material from 3 tubes to 2 tubes. Rinse emptied tubes in the same way as described in step 8 and bring each tube to 50 mL with 0.1% SDS solution.
12) Centrifuge at 300 × *g* for 30 min at 26°C using a benchtop centrifuge.
13) Remove the supernatant leaving at least 5 mL liquid above the pellet.
14) Combine the material from 2 tubes to 1 tube. Rinse emptied tube in the say way as described in step 8 and bring to 50 mL with 0.1% SDS solution.
15) Centrifuge at 300 × *g* for 30 min at 26°C using a Bench top centrifuge.
16) Remove the supernatant leaving 4 mL liquid above the pellet.
17) Resuspend the material by homogenizing with 20 strokes of a Teflon-glass Dounce homogenizer.
18) Pass the homogenate through 38 um Nylon mesh, collect flow through into a 50 mL tube. Rinse homogenizer tube with 1 mL 0.1% SDS solution, pass through the same filter and collect flow through. Repeat 2 more times.
19) The above procedure typically yields ∼7–7.5 mL crude plaque materials from 50 g of gray matter. Store sample at 4°C overnight.

#### 3.2.3. Plaque enrichment by gradient centrifugation (estimated time: 4 h)

1) Prepare a linear sucrose gradient in a 38.5 mL ultracentrifuge tube. First add 5 ml of 1.8 M sucrose solution to the bottom, then carefully add 8 mL of 1.6 M sucrose solution on top of the 1.8 M sucrose solution. Thereafter, add 8 mL of 1.4 M sucrose solution and 7 mL of 1.2 M sucrose solution on top of the former. Carefully apply 7.5 mL crude plaque suspension to the top of the gradient.
2) Centrifuge in an SW28 rotor at 72,000 × *g* for 60 min at 26°C.
  Note: Ensure the centrifuge is set at 26°C as SDS will crystallize at lower temperature.
3) Beginning at the top collect each layer and transfer to individual 50 mL tubes.
  Note: Typically most amyloid plaque are found in the 1.6 M sucrose layer. Nonetheless, we recommend collecting materials from all layers for further analysis.
4) Dilute each layer sample 1:5 with 0.1% SDS in 150 mM NaCl. Centrifuge at 300 × *g* for 30 min at 26°C, then transfer each pellet to a 1.5 mL Protein-low bind Eppendorf tube.
5) Centrifuge at 12,000 × *g* and room temperature for 10 min. Discard supernatant and keep 100 uL liquid above the pellet. Add 1 mL filtered MQ water into each tube and vortex thoroughly.
6) Repeat step 6 one more time.
7) Centrifuge at 12,000 × *g* and room temperature for 10 min. Discard supernatant and resuspend pelleted plaques with 100 uL of MQ water. Take 10 uL of this material, stain with 0.2% Congo red and visualize using polarized microscopy (see section 2.2. Equipment).
8) Freeze the remaining material and lyophilize.
9) To concentrate lyophilizate at the bottom of the tube centrifuge at 12,000 × *g* and room temperature for 10 min.
  Note: The weight of isolated plaques can be estimated by subtracting the weight of pre-weighed empty tubes from the weight of tubes containing plaques.

#### 3.2.4. Plaque solubilization and Aβ purification (estimated time: 1 day)

1) Add 1.2 mL of 88% formic acid to each vial of lyophilized plaques, vortex and incubate at room temperature overnight, e.g., 12–14 h, with gentle agitation.
  Note: Do not incubate plaques in formic acid longer than 24 h.
2) Centrifuge at 12,000 × *g* for 15 min at room temperature and carefully transfer the upper 90% of supernatant into a 1.5 mL Protein-low bind Eppendorf tube.
3) Equilibrate Superdex 75 10/300 GL size exclusion column (SEC) in 50 mM Ammonium Bicarbonate, pH 8.5 at 0.4 mL/min.
4) Inject 1 mL of blue dextran solution and collect 0.5 mL fractions. The peak fraction containing blue dextran is designated as “void volume” and labeled as Fx0. Equilibrate system with 2 column volumes (CV) of Mobile Phase solution.
5) Inject 1 mL of Gel filtration standard (Bio-Rad) and calibrate standards elution by using UV detector. Equilibrate system with 2 CVs of Mobile Phase solution.
6) Inject 1 mL solubilized plaque material and collect 0.5 mL fractions from Fx-1 to 17. Take 5 uL of each fraction and measure Aβ by Western Blot as described previously (Brinkmalm et al., 2019). Store remainder of the fractions at −80°C pending further analysis.

### 3.3. Application of human iPSC-derived neurons and live-cell imaging to evaluate neuritoxicity induced by soluble Aβ

#### 3.3.1. Generation of induced human neurons (estimated time: 3 weeks)

1) Plate human iPS cells on Matrigel coated 12-well plate with 3.8 × 10^5^ per well in mTeSR media.
2) On the day of viral infection, prepare 1 mL of fresh mTeSR media containing pTet-O-NGN2-puro lentivirus, Tet-O-FUW-eGFP lentivirus and Fudelta GW-rtTA lentivirus. Remove cell culture medium and add lentivirus-containing medium.
Note: Lentiviruses were prepared using the plasmids from Addgene (#52047, #30130, and #19780) as showed previously (Zhang et al., 2013). The efficiency of lentivirus infection to different iPS cells may be different, thus we recommend testing their infectivity and use the most efficient multiplicity of infection (MOI) in further experiments. Puromycin is used to select infected iPS cells, while the applicable concentration may differ from cell line to cell line. Thus, we recommend conducting a small induction trial (see steps 4–11) to test puromycin selection and neuron differentiation.
3) After 24 h, remove lentivirus-containing medium. Dissociate cells with Accutase and resuspend with mTeSR supplemented with Y-27632. Transfer cells to 10 cm dishes and feed with fresh mTeSR media every day. When cells reach >80% confluent, split for expansion or freeze down.
  Note: To avoid batch effect of lentivirus infection, we recommend freezing down a large number of cryovials of transduced iPS cells. Transduced iPS cells can be passaged no more than 10 times to maintain high efficiency of neuron differentiation.
4) Plate 4 × 10^6^ transduced iPS cells using mTeSR with Y-27632 on Matrigel coated 10 cm dish. Feed with 7 mL fresh mTeSR media every day until cells reach 50% confluence.
5) On day 1, remove cell culture medium and add 7 mL KSR media containing with 2 μg/mL Doxycycline (iN induction medium A).
  Note: At this stage, Ngn2 and GFP expression are induced.
6) On day 2, replace cell culture medium with KSR:N2B (1:1) media containing with 5 μg/mL Puromycin and 2 μg/mL Doxycycline (iN induction medium B).
  Note: At this stage, cells without Ngn2/Puromycin resistance would die off.
7) On day 3, replace cell culture medium with N2B media containing with 1% B27, 5 μg/mL Puromycin and 2 μg/mL Doxycycline (iN induction medium C).
8) On day 4, split cells for neuron differentiation or freeze down. For freezing down, wash cells with warm PBS and detach with Accutase. Resuspend with freezing medium and store in liquid nitrogen.
  Note: The neuronal differentiation efficiency may differ from batch to batch. To minimize batch effect, we recommend freezing down a large down a large number of cryovials of iN4 (induced neuron at day 4) and using the same batch of cells in one study.
9) For neuron differentiation, plate 1.5 × 10^4^ iN4 cells/cm^2^ using iN culture media with Y-27362 onto Matrigel coated cell culture vessels. Feed cells with iN culture media.
  Note: Select appropriate cell culture vessels according to practical requirements. When changing iN culture medium, we recommend removing half of the medium and add the same volume of fresh medium. Ara-C is optional if divided cells are seen in the iN culture.
10) Change iN culture media every 3∼4 days. In our preliminary experiments, iN cells mature at iN21, i.e., 17 days after plating, and can be used throughout a time window (approximately a week).

#### 3.3.2. Assessment of neurotoxic activity of Aβ-containing brain extract using live-cell imaging (estimated time: 3–4 days)

1) For Aβ toxicity measurement using live-cell imaging system, plate 5 × 10^3^ iN4 cells onto Matrigel coated 96-well plate. Feed cells with iN culture medium every 3–4 days.
  Note: To minimize the effect of evaporation cell culture medium from neurons, we recommend plating iN cells into the inner 60 wells of 96-well plate and adding PBS into the outer wells.
2) Approximately 7 h prior to treatment, place cell culture plates into IncuCyte live-cell imaging instrument. Set up scanning procedure following manufacturer’s instructions. For long-term recording, schedule repeating scans every 2 h for up to 96 h.
  Note: We recommend recording iN cells 7 h prior to treatment. This allows acquisition of three baseline scans which provide a solid reference for time course studies. During this period Aβ-containing human brain extracts are buffer-exchanged into neurobasal medium supplemented with B27/GlutaMAX.
3) Buffer exchange is accomplished using a 5 mL HiTrap desalting column and a peristaltic pump. Wash the column with 25 ml of 20% Ethanol and then 25 mL of filtered MQ water. Then equilibrate with 25 mL of neurobasal medium supplemented with B27/GlutaMAX.
4) Apply 0.5 mL of human brain extract using a 1 mL syringe at a flow rate of ∼1 mL/min. Elute proteins with iN culture medium and collect eight 0.5 mL fractions.
5) Remove 50 μL of each fraction and measure Aβ concentration by ELISA. Pool fractions 4 and 5 (total volume 1 mL) and use for further toxicity assays.
  Note: Prior studies indicate that fractions 4 and 5 consistently contain the peak Aβ fractions and exclude low molecular weight brain-derived compounds. Confirmatory ELISA analysis is done on the day after iN cell treatment.
6) During the time after the 3rd baseline scan, remove cell culture plates from IncuCyte instrument. Remove approximately half of cell culture medium leaving ∼100 μL in each well. Then add 50 μL of fresh iN culture medium and 50 μL of buffer-exchanged human brain extract. This results in a 1:8 dilution of the original brain extract.
  Note: If the scan interval is 2 h, we should be cautious of the time window that allows us to complete the sample application. In fact, the scan interval is optional depending on practical requirements and manipulation feasibility.
7) Place treated cells into IncuCyte instrument and continue recording for 3∼4 days.
8) When experiment is completed, select representative images from different wells to a new NeuroTrack image collection. Launch New Processing Definition and adjust parameters following manufacturer’s instructions to define neurite processes and cell bodies based on phase contrast images. Once the training is completed, save the processing definition as a confirmed analysis template.
  Note: Typical settings were: Segmentation Mode–Brightness; Segmentation Adjustment–1.2; Cell body cluster filter–minimum 500 μm^2^; Neurite Filtering–Best; Neurite sensitivity–0.4; Neurite Width–2 μm.
9) Launch new analysis job to the recorded database of the whole plate using the above confirmed analysis template.
  Note: The training of processing definition can be conducted using the baseline scan data. Thus, we can launch real-time analysis job and monitor the scheduled scanning to the whole cell culture plate. Alternatively, an analysis job can be launched when the experiment is completed or even during the scheduled scanning.
10) Export Metric Graph/data and quantify neurite length and number of branch points. The neurotoxicity of Aβ is determined to the average value measured during the 6 h period prior to sample addition.

## 4. Data analysis and statistical tests

Figures showing MSD Aβ immunoassay and live-cell IncuCyte imaging data are representative of at least three independent experiments. Differences between groups were tested with one-way analysis of variance (ANOVA) with Bonferroni *post-hoc* tests or Student’s *t*-tests using GraphPad Prism 8.

## 5. Anticipated results

We have used the above protocols to isolate and characterize Aβ from multiple AD brains–but here we show only a few representative cases. The amount of Aβ extracted by Method I (gently soaking minced tissue in aqueous buffer–S extract) and Method II (mechanically homogenizing tissue–H extract) was measured using highly sensitive Aβ immunoassays. Typically, S extracts contain substantially less Aβ than H extracts (Figure 1; Hong et al., 2018). Since Aβ does not distribute equally across brain regions or in different sections from the same brain region, we used large amounts of (∼20 g) tissue from each brain. To directly compare H extracts and S extracts, chopped tissue chunks were mixed thoroughly and half of the material was used to prepare H or S extracts. Analysis of at least 10 AD brains revealed that freely diffusible Aβ can be extracted from brain tissue without the need of mechanical disruption.

Human brain extracts may vary in their content of bioactive small molecules such as neurotransmitters and/or pharmacologic agents, thus it is important to take precautions to remove any such material prior to assessing bioactivity. For this reason we dialyzed brain extracts immediately after preparation. For routine analysis we measured glutamate levels in human brain extracts before and after dialysis. We found that both H and S extracts contained ∼300 μM of glutamate, and that >90% of the starting concentration of glutamate was removed by three rounds of dialysis (Figure 2). Prior studies revealed that the low level of remaining glutamate did not adversely affect electrophysiological measurement of hippocampal LTP or the morphology of iNs (Wang et al., 2017; Hong et al., 2018). Moreover, further buffer exchange, such as that done prior to addition of extracts to iNs is expected to further deplete glutamate and other low molecular compounds.

**FIGURE 3:**
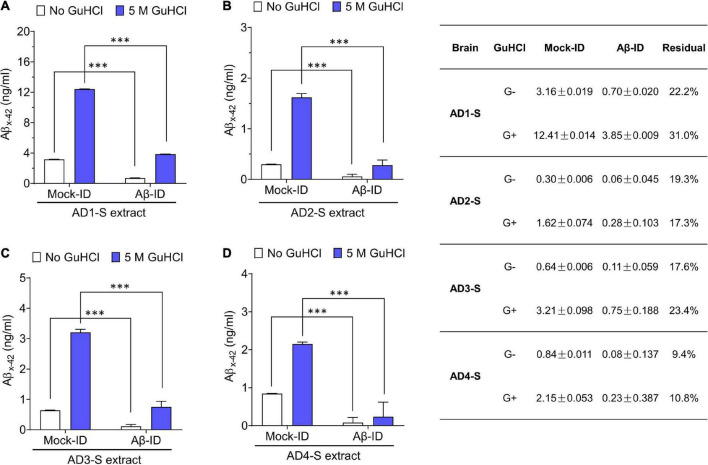
Pan anti-Aβ polyclonal antibody S97 readily depletes Aβ from human brain extracts. **(A–D)** Samples were immunodepleted with S97 (Aβ-ID) or PIS (Mock-ID) conjugated to Protein A Sepharose beads (PAS). Aβ content in Mock-ID and Aβ-ID of S extracts from four AD brains was measured using the Aβx-42 immunoassay ± pre-treatment with 5 M GuHCl. S97 effectively immunodepleted Aβx-42 from S extracts of four AD brains. Table in the right panel indicates the values measured by ELISA. G– and G+ denote no GuHCl and 5 M GuHCl, respectively. Residual Aβ is presented as the ratio of Aβ-ID vs. Mock-ID. Values were shown as mean ± SD. Treatments were examined by one-way ANOVA and significant differences are denoted as ^***^p < 0.001.

**FIGURE 4:**
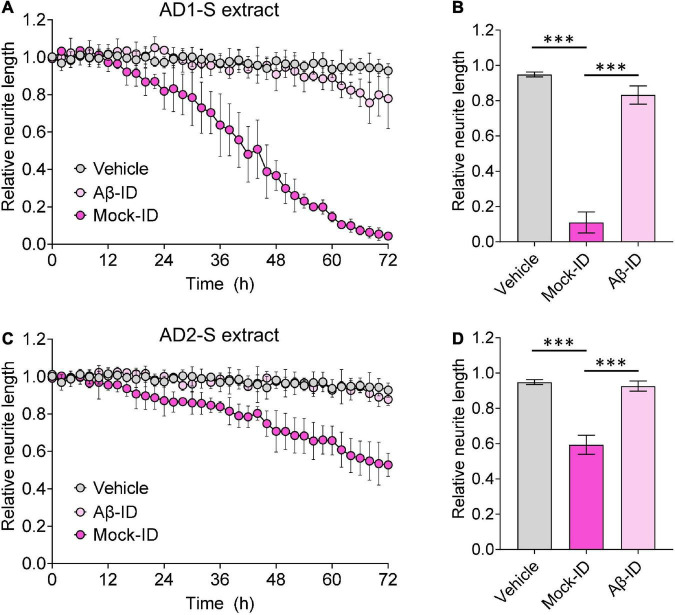
Aqueous AD brain extracts impairs neuritic integrity of iNs in a manner that requires Aβ. Mock-ID’d and Aβ-ID’d S extracts of two AD brains were used to treat iNs at dilution of 1:4, and cells imaged using IncuCyte live-cell imaging instrument for 72 h. NeuroTrack-identified neurite length was measured and normalized to baseline (6 h recordings prior to sample addition) at each interval after addition of sample. **(A,C)** Time-course plots show that Mock-ID’d S extracts from 2 AD brains cause neurotoxicity compared to vehicle. **(B,D)** Histogram plots of normalized neurite length (mean values ± SD) are derived from the last 6 h of the traces shown in panels **(A,C)**. Treatments were examined by one-way ANOVA and significant differences are denoted as ^***^p < 0.001. Panel **(B)** is a reproduction of a figure previously published in Brinkmalm et al. (2019).

To determine whether the neuritotoxicity induced by AD brain extracts was mediated by Aβ, we removed Aβ using a pan anti-Aβ polyclonal antibody, (e.g., S97 conjugated to PAS beads). S97 readily removes >70% of Aβx-42 from S extracts (Figure 3). Consistent with our previous studies, Mock-ID’d S extracts caused a time-dependent decrease in neurite length, while the Aβ-ID’d S extracts did not (Figures 4A, C). These observations indicate that the neuritotoxicity induced by S extracts is mediated by Aβ (Figures 4B, D).

**FIGURE 5:**
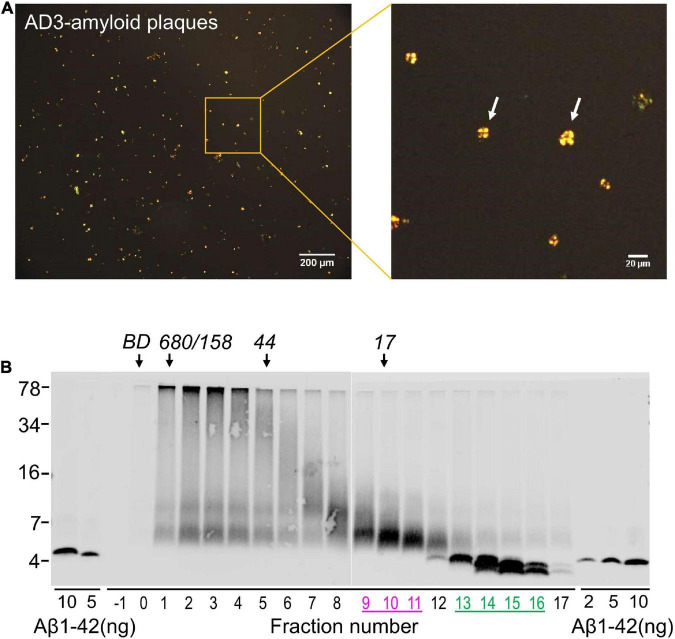
Solubilized amyloid plaques contain Aβ dimers and monomers. **(A)** Amyloid plaques were isolated from AD brain and characterized by Congo red staining. **(B)** Congo red-positive amyloid plaques were dissolved in formic acid, chromatographed on SEC and used for western blot with 4G8. The elution of Blue dextran (BD) and globular protein standards is indicated by downward arrows and SDS-PAGE molecular weight standards are on the left. Synthetic Aβ1–42 was used as control for Western blotting efficiency.

**FIGURE 6:**
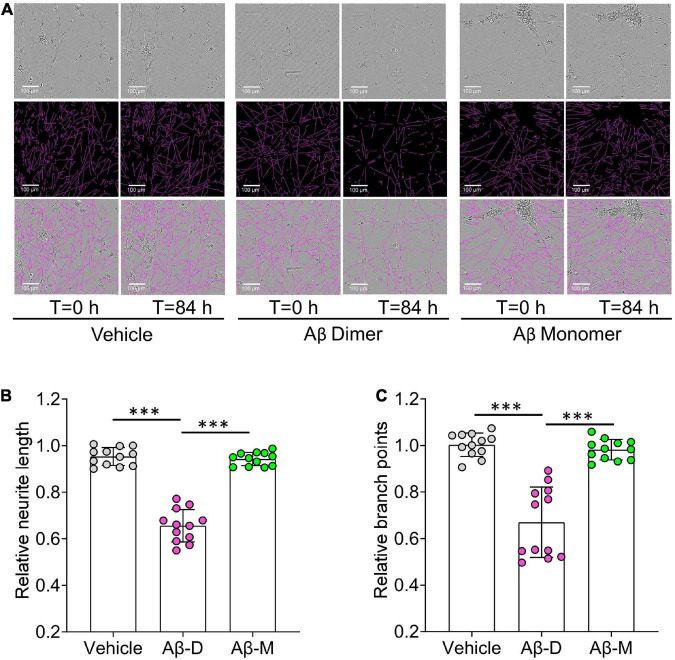
Human brain derived Aβ dimers induce neuritic dystrophy when applied to iNs. **(A)** Mature human iPSC-induced neurons (iNs) were treated with vehicle, 22 nM of AD brain derived Aβ dimers (Aβ Dimer) or monomers (Aβ Monomer), and cells imaged using IncuCyte live-cell imaging instrument for 84 h. Phase contrast images (top panel) were used to identify neurites (middle panel) using NeuroTrack software. Identified neurites (purple) are shown on the phase contrast images (bottom panel). Scare bars are 100 μm. **(B,C)** Each well of iNs was imaged for 6 h prior to addition of sample used as baseline. Relative neurite length and branch points at *T* = 0 and 84 h were normalized to corresponding baselines. Treatments were examined by one-way ANOVA and significant differences are denoted as ^***^p < 0.001.

Using an established method for isolating amyloid plaques from human cortex, we obtained microgram quantities of Congo red-positive plaques (Figure 5A) which were then solubilized in formic acid and fractionated using size exclusion chromatography. Fractions containing dimer (Fx9-11) and monomer (Fx13-16) were identified (Figure 5B) and respective fractions pooled and their effect on iNs assessed using our IncuCyte live cell imaging paradigm. When applied to iNs at a concentration of 100 ng/mL, Aβ dimers potently disrupted neurite, whereas even at higher concentration Aβ monomers had no effect (Figure 6A). Specifically, over the last 12 h of recording Aβ dimers caused a significant decrease in neurite length and branch points both relative to pre-treatment values, and compared to vehicle (*p* < 0.001) or monomers (*p* < 0.001) (Figures 6B, C). Prior analysis indicates that SEC isolated monomer does not readily form dimers under conditions used to study bioactivity (Brinkmalm et al., 2019).

Although not shown in the current study we have previously used brains from non-Alzheimer subjects as a control (Shankar et al., 2008; Wang et al., 2017, 2022; Hong et al., 2018; Jin et al., 2018) and found they lack bioactivity. We recommend the use of such controls when testing material produced by Method I or Method II, however, since the use of Method III is primarily to compare plaque derived monomer vs. dimer and AD brain is the most abundant and biologically relevant source of plaques the use of difficult to access control brain is not be required.

## 6. Discussion

In contrast to the thousands of studies that have used synthetic Aβ, rodent neurons, or irrelevant cell lines and measures of cell death (Glabe, 2008; Yankner and Lu, 2009; Benilova et al., 2012; Hampel et al., 2021), we pioneered an approach to measure bioactivity utilizing Aβ from human brain applied to human neurons and a readout directly relevant to the human disease. It is well known that Aβ occurs in many forms and there is controversy about which of these mediate disease. Here, we describe protocols to enable the study of different populations of Aβ. Method I involves soaking minced brain tissue in aqueous buffer and allows the isolation of readily diffusible Aβ with high bioactivity. Method II utilizes homogenization, the product of which contains the same species produced by Method I, plus a large amount of inactive species released by mechanical disruption. Comparison of extracts produced by these methods should allow a greater understanding of the molecular properties of active vs. inactive Aβ. Initial studies comparing Aβ containing extracts prepared using Method I vs. Method II revealed that S extracts contained much lower levels of Aβ than H extracts, while both equivalently block long-term potentiation and impair neuritic integrity (Hong et al., 2018). Moreover, when pellets from S extracts were homogenized and clarified to produce H2 extracts, H2 extracts were found to contain Aβ at levels comparable to H extracts, but H2 extracts were not bioactive. These results demonstrate that Method II enables the isolation of readily solubilized forms of Aβ the majority of which are inactive. Nonetheless, when screening for agents to nullify bioactive Aβ, H extracts provide a stringency tool to assess specificity.

Method III enables isolation of highly pure Aβ dimers and monomers from amyloid plaques. While we have shown dimers to have disease relevant activity (Shankar et al., 2008; Brinkmalm et al., 2019; Zott et al., 2019), certain reports indicate that Aβ monomer has beneficial effects (Plant et al., 2006; Puzzo et al., 2008; Sturchio et al., 2022; Zhou et al., 2022). Utilization of Method III should allow a direct comparison of the activities of monomers and dimers, and the design of agents that would inhibit the adverse effects of dimers without impairing the potentially beneficial effects of monomers. One major advantage of using plaque derived Aβ vs. synthetic Aβ is the fact that brain derived Aβ is molecularly heterogeneous such that recapitulating this complexity with synthetic peptides is challenging. Specifically, mass spectrometric analysis of plaque derived monomer from six human AD brains, identified 36 different primary Aβ structures (Brinkmalm et al., 2019). Dimers are even more complex, both with regard to the component monomers that contribute to dimers and the linkages that hold dimers together (Brinkmalm et al., 2019). In a separate study we showed that human brain derived Aβ dimers induce neuronal hyperactivation at concentrations estimated to be <50 nM, whereas dimers formed from synthetic Aβ dimer required concentrations of 500 nM to achieve a comparable effect (Zott et al., 2019). It seems reasonable to speculate that the molecular complexity of brain derived dimers contributes to their much higher specific activity relative to synthetic dimers. Although not a focus of this report, we would be remise, if we did not note that Method III also describes the isolation plaques which without solubilization could prove useful for generation of antibodies and other possible studies.

In future studies, CRISPR-based approaches could be employed to interrogate pathways of both Aβ toxicity and synaptogenic effects. Thus, the combination of the three extraction methods described and the further adaptation of our bioassay should enable a deeper understanding of the positive and negative activities of Aβ and may facilitate discovery of agents highly specific for the most noxious forms of Aβ.

Limitations of our approach include the requirement for large amounts of postmortem brain tissue, and relatively long duration experiments. Typically, preparation and characterization of brain extracts and/or plaque-derived Aβ takes on the order of 2 weeks. The bioassay is medium throughput and requires data collection periods of 2–4 days. To exclude artifacts arising due to postmortem delays and/or freeze-thawing of tissue, for certain studies it will be important to investigate the use of fresh biopsy tissue. Nonetheless, other investigators have already employed our experimental paradigms (Sideris et al., 2021; Emin et al., 2022; Hsieh et al., 2022; Liu et al., 2022; Stern et al., 2022) and we expect that these protocols will be further exploited. For instance to examine the bioactivity of tau and α-synuclein (Corbett et al., 2020). It is also important to point out that Aβ constitutes only a small fraction of the total protein produced by Method I and Method II and that certain AD brain extracts mediate toxicity independent of Aβ (Ondrejcak et al., 2018; Wang et al., 2022). Here, we focused on brain extracts for which toxicity could be abolished by immunodepletion of Aβ. In contrast, the Aβ species produced by Method III are highly pure and free from other detectable proteins (Hong et al., 2018).

## Data availability statement

The original contributions presented in this study are included in the article, further inquiries can be directed to the corresponding author.

## Ethics statement

This study was reviewed and approved by Partners IRB. Human specimens were obtained from the Massachusetts ADRC Neuropathology Core, Massachusetts General Hospital and used in accordance with the Partners Institutional Review Board (Protocol: Walsh BWH 2011). Written informed consent was obtained from the individual(s) to participate in this study.

## Author contributions

DMW conceived the research. DMW and WH designed the protocols and wrote the manuscript. WH performed the biochemical and IncuCyte experiments, analyzed the data, and prepared the figures. WL cultured iPSC-derived neurons and assisted with biochemical experiments. WL and AD isolated and characterized the amyloid plaques. TY-P provided the viral-infected iPS cells. All authors critically reviewed the manuscript.

## References

[B1] Allsop D. Landon M. Kidd M. (1983). The isolation and amino acid composition of senile plaque core protein. *Brain Res.* 259 348–352. 10.1016/0006-8993(83)91273-8 6824945

[B2] Benilova I. Karran E. De Strooper B. (2012). The toxic Abeta oligomer and Alzheimer’s disease: an emperor in need of clothes. *Nat. Neurosci.* 15 349–357. 10.1038/nn.3028 22286176

[B3] Brinkmalm G. Hong W. Wang Z. Liu W. O’malley T. T. Sun X. (2019). Identification of neurotoxic cross-linked amyloid-beta dimers in the Alzheimer’s brain. *Brain* 142 1441–1457. 10.1093/brain/awz066 31032851 PMC6487330

[B4] Brody D. L. Jiang H. Wildburger N. Esparza T. J. (2017). Non-canonical soluble amyloid-beta aggregates and plaque buffering: controversies and future directions for target discovery in Alzheimer’s disease. *Alzheimers Res. Ther.* 9:62. 10.1186/s13195-017-0293-3 28818091 PMC5561579

[B5] Budd Haeberlein S. Aisen P. S. Barkhof F. Chalkias S. Chen T. Cohen S. (2022). Two Randomized Phase 3 Studies of Aducanumab in Early Alzheimer’s Disease. *J. Prev. Alzheimers Dis.* 9 197–210. 10.14283/jpad.2022.30 35542991

[B6] Corbett G. T. Wang Z. Hong W. Colom-Cadena M. Rose J. Liao M. (2020). PrP is a central player in toxicity mediated by soluble aggregates of neurodegeneration-causing proteins. *Acta Neuropathol.* 139 503–526. 10.1007/s00401-019-02114-9 31853635 PMC7035229

[B7] Emin D. Zhang Y. P. Lobanova E. Miller A. Li X. Xia Z. (2022). Small soluble alpha-synuclein aggregates are the toxic species in Parkinson’s disease. *Nat. Commun.* 13:5512. 10.1038/s41467-022-33252-6 36127374 PMC9489799

[B8] Enya M. Morishima-Kawashima M. Yoshimura M. Shinkai Y. Kusui K. Khan K. (1999). Appearance of sodium dodecyl sulfate-stable amyloid beta-protein (Abeta) dimer in the cortex during aging. *Am. J. Pathol.* 154 271–279. 10.1016/S0002-9440(10)65273-X 9916941 PMC1853431

[B9] Glabe C. G. (2008). Structural classification of toxic amyloid oligomers. *J. Biol. Chem.* 283 29639–29643. 10.1074/jbc.R800016200 18723507 PMC2573087

[B10] Glenner G. G. Wong C. W. (1984). Alzheimer’s disease: initial report of the purification and characterization of a novel cerebrovascular amyloid protein. *Biochem. Biophys. Res. Commun.* 120 885–890. 10.1016/S0006-291X(84)80190-4 6375662

[B11] Hampel H. Hardy J. Blennow K. Chen C. Perry G. Kim S. H. (2021). The Amyloid-beta pathway in Alzheimer’s disease. *Mol. Psychiatry* 26 5481–5503. 10.1038/s41380-021-01249-0 34456336 PMC8758495

[B12] Hong W. Wang Z. Liu W. O’malley T. T. Jin M. Willem M. (2018). Diffusible, highly bioactive oligomers represent a critical minority of soluble Abeta in Alzheimer’s disease brain. *Acta Neuropathol.* 136 19–40. 10.1007/s00401-018-1846-7 29687257 PMC6647843

[B13] Hsieh Y. C. Negri J. He A. Pearse R. V. II Liu L. Duong D. M. (2022). Elevated ganglioside GM2 activator (GM2A) in human brain tissue reduces neurite integrity and spontaneous neuronal activity. *Mol. Neurodegener.* 17:61. 10.1186/s13024-022-00558-4 36131294 PMC9494921

[B14] Jack C. R. Jr. Wiste H. J. Vemuri P. Weigand S. D. Senjem M. L. Zeng G. (2010). Brain beta-amyloid measures and magnetic resonance imaging atrophy both predict time-to-progression from mild cognitive impairment to Alzheimer’s disease. *Brain* 133 3336–3348. 10.1093/brain/awq277 20935035 PMC2965425

[B15] Jin M. O’Nuallain B. Hong W. Boyd J. Lagomarsino V. N. O’M alley T. T. (2018). An in vitro paradigm to assess potential anti-Abeta antibodies for Alzheimer’s disease. *Nat. Commun.* 9:2676. 10.1038/s41467-018-05068-w 29992960 PMC6041266

[B16] Kuo Y. M. Emmerling M. R. Vigo-Pelfrey C. Kasunic T. C. Kirkpatrick J. B. Murdoch G. H. (1996). Water-soluble Abeta (N-40, N-42) oligomers in normal and Alzheimer disease brains. *J. Biol. Chem.* 271 4077–4081. 10.1074/jbc.271.8.4077 8626743

[B17] Kuo Y. M. Emmerling M. R. Woods A. S. Cotter R. J. Roher A. E. (1997). Isolation, chemical characterization, and quantitation of A beta 3-pyroglutamyl peptide from neuritic plaques and vascular amyloid deposits. *Biochem. Biophys. Res. Commun.* 237 188–191. 10.1006/bbrc.1997.7083 9266855

[B18] Lau H. H. C. Ingelsson M. Watts J. C. (2021). The existence of Abeta strains and their potential for driving phenotypic heterogeneity in Alzheimer’s disease. *Acta Neuropathol.* 142 17–39. 10.1007/s00401-020-02201-2 32743745

[B19] Liu L. Kwak H. Lawton T. L. Jin S. X. Meunier A. L. Dang Y. (2022). An ultra-sensitive immunoassay detects and quantifies soluble Abeta oligomers in human plasma. *Alzheimers Dement.* 18 1186–1202. 10.1002/alz.12457 34550630 PMC8938295

[B20] Masters C. L. Simms G. Weinman N. A. Multhaup G. Mcdonald B. L. Beyreuther K. (1985). Amyloid plaque core protein in Alzheimer disease and down syndrome. *Proc. Natl. Acad. Sci. U.S.A.* 82 4245–4249. 10.1073/pnas.82.12.4245 3159021 PMC397973

[B21] Mc Donald J. M. O’malley T. T. Liu W. Mably A. J. Brinkmalm G. Portelius E. (2015). The aqueous phase of Alzheimer’s disease brain contains assemblies built from approximately 4 and approximately 7 kDa Abeta species. *Alzheimers Dement.* 11 1286–1305. 10.1016/j.jalz.2015.01.005 25846299 PMC4592782

[B22] McDade E. Bateman R. J. (2017). Stop Alzheimer’s before it starts. *Nature* 547 153–155. 10.1038/547153a 28703214

[B23] Mintun M. A. Lo A. C. Duggan Evans C. Wessels A. M. Ardayfio P. A. Andersen S. W. (2021). Donanemab in early Alzheimer’s Disease. *N. Engl. J. Med.* 384 1691–1704. 10.1056/NEJMoa2100708 33720637

[B24] Mukherjee S. Perez K. A. Lago L. C. Klatt S. Mclean C. A. Birchall I. E. (2021). Quantification of N-terminal amyloid-beta isoforms reveals isomers are the most abundant form of the amyloid-beta peptide in sporadic Alzheimer’s disease. *Brain Commun.* 3:fcab028. 10.1093/braincomms/fcab028 33928245 PMC8062259

[B25] Ondrejcak T. Klyubin I. Corbett G. T. Fraser G. Hong W. Mably A. J. (2018). Cellular Prion Protein Mediates the Disruption of Hippocampal Synaptic Plasticity by Soluble Tau In Vivo. *J. Neurosci.* 38 10595–10606. 10.1523/JNEUROSCI.1700-18.2018 30355631 PMC6290298

[B26] Plant L. D. Webster N. J. Boyle J. P. Ramsden M. Freir D. B. Peers C. (2006). Amyloid beta peptide as a physiological modulator of neuronal ‘A’-type K+ current. *Neurobiol. Aging* 27 1673–1683. 10.1016/j.neurobiolaging.2005.09.038 16271805

[B27] Puzzo D. Privitera L. Leznik E. Fa M. Staniszewski A. Palmeri A. (2008). Picomolar amyloid-beta positively modulates synaptic plasticity and memory in hippocampus. *J. Neurosci.* 28 14537–14545. 10.1523/JNEUROSCI.2692-08.2008 19118188 PMC2673049

[B28] Rafii M. S. Sperling R. A. Donohue M. C. Zhou J. Roberts C. Irizarry M. C. (2022). The AHEAD 3-45 study: design of a prevention trial for Alzheimer’s disease. *Alzheimers Dement.* [Epub ahead of print]. 10.1002/alz.12748 35971310 PMC9929028

[B29] Roher A. E. Chaney M. O. Kuo Y. M. Webster S. D. Stine W. B. Haverkamp L. J. (1996). Morphology and toxicity of Abeta-(1-42) dimer derived from neuritic and vascular amyloid deposits of Alzheimer’s disease. *J. Biol. Chem.* 271 20631–20635. 10.1074/jbc.271.34.20631 8702810

[B30] Selkoe D. J. Hardy J. (2016). The amyloid hypothesis of Alzheimer’s disease at 25 years. *EMBO Mol. Med.* 8 595–608. 10.15252/emmm.201606210 27025652 PMC4888851

[B31] Sevigny J. Chiao P. Bussiere T. Weinreb P. H. Williams L. Maier M. (2017). Addendum: The antibody aducanumab reduces Abeta plaques in Alzheimer’s disease. *Nature* 546:564. 10.1038/nature22809 28640269

[B32] Shankar G. M. Li S. Mehta T. H. Garcia-Munoz A. Shepardson N. E. Smith I. (2008). Amyloid-beta protein dimers isolated directly from Alzheimer’s brains impair synaptic plasticity and memory. *Nat. Med.* 14 837–842. 10.1038/nm1782 18568035 PMC2772133

[B33] Shinkai Y. Yoshimura M. Morishima-Kawashima M. Ito Y. Shimada H. Yanagisawa K. (1997). Amyloid beta-protein deposition in the leptomeninges and cerebral cortex. *Ann. Neurol.* 42 899–908. 10.1002/ana.410420612 9403483

[B34] Sideris D. I. Danial J. S. H. Emin D. Ruggeri F. S. Xia Z. Zhang Y. P. (2021). Soluble amyloid beta-containing aggregates are present throughout the brain at early stages of Alzheimer’s disease. *Brain Commun.* 3:fcab147. 10.1093/braincomms/fcab147 34396107 PMC8361392

[B35] Stern A. M. Liu L. Jin S. Liu W. Meunier A. L. Ericsson M. (2022). A calcium-sensitive antibody isolates soluble amyloid-beta aggregates and fibrils from Alzheimer’s disease brain. *Brain* 145 2528–2540. 10.1093/brain/awac023 35084489 PMC9337809

[B36] Sturchio A. Dwivedi A. K. Malm T. Wood M. J. A. Cilia R. Sharma J. S. (2022). High soluble amyloid-beta42 predicts normal cognition in amyloid-positive individuals with Alzheimer’s disease-causing mutations. *J. Alzheimers Dis.* 90 333–348. 10.3233/JAD-220808 36120786 PMC9661329

[B37] Suzuki N. Iwatsubo T. Odaka A. Ishibashi Y. Kitada C. Ihara Y. (1994). High tissue content of soluble beta 1-40 is linked to cerebral amyloid angiopathy. *Am. J. Pathol.* 145 452–460. 8053502 PMC1887395

[B38] Swanson C. Dhadda S. Irizarry M. Kanekiyo M. Li D. Koyama A. (2021a). An assessment of the clinical effects, the correlation of plasma Ab ratio with changes in brain amyloid PET SUVR, and safety from the core and open label extension of the Phase 2 proof-of-concept study, BAN2401-G000-201, in subjects with early Alzheimer’s disease. 13. 10.1002/alz.057760

[B39] Swanson C.. Zhang Y. Dhadda S. Wang J. Kaplow J. Lai R. Y. K. (2021b). A randomized, double-blind, phase 2b proof-of-concept clinical trial in early Alzheimer’s disease with lecanemab, an anti-Abeta protofibril antibody. *Alzheimers Res. Ther.* 13:80. 10.1186/s13195-021-00813-8 33865446 PMC8053280

[B40] van Dyck C. H. Swanson C. J. Aisen P. Bateman R. J. Chen C. Gee M. (2022). Lecanemab in early Alzheimer’s disease. *N. Engl. J. Med.* 88 9–21. 10.1056/NEJMoa2212948 36449413

[B41] Wang Z. Jackson R. J. Hong W. Taylor W. M. Corbett G. T. Moreno A. (2017). Human brain-derived abeta oligomers bind to synapses and disrupt synaptic activity in a manner that requires APP. *J. Neurosci.* 37 11947–11966. 10.1523/JNEUROSCI.2009-17.2017 29101243 PMC5719975

[B42] Wang Z. Jin M. Hong W. Liu W. Reczek D. Lagomarsino V. N. (2022). Learnings about Ab from human brain recommend the use of a live-neuron bioassay for the discovery of next generation Alzheimer’s disease immunotherapeutics. *Acta Neuropathol. Commun*. (In press). 10.1186/s40478-023-01511-2 PMC10007750 36899414

[B43] Welander H. Franberg J. Graff C. Sundstrom E. Winblad B. Tjernberg L. O. (2009). Abeta43 is more frequent than Abeta40 in amyloid plaque cores from Alzheimer disease brains. *J. Neurochem.* 110 697–706. 10.1111/j.1471-4159.2009.06170.x 19457079

[B44] Wildburger N. C. Esparza T. J. Leduc R. D. Fellers R. T. Thomas P. M. Cairns N. J. (2017). Diversity of amyloid-beta proteoforms in the Alzheimer’s disease brain. *Sci. Rep.* 7:9520. 10.1038/s41598-017-10422-x 28842697 PMC5572664

[B45] Yang T. Li S. Xu H. Walsh D. M. Selkoe D. J. (2017). Large soluble oligomers of amyloid beta-protein from Alzheimer brain are far less neuroactive than the smaller oligomers to which they dissociate. *J. Neurosci.* 37 152–163. 10.1523/JNEUROSCI.1698-16.2016 28053038 PMC5214627

[B46] Yankner B. A. Lu T. (2009). Amyloid beta-protein toxicity and the pathogenesis of Alzheimer disease. *J. Biol. Chem.* 284 4755–4759. 10.1074/jbc.R800018200 18957434 PMC2643502

[B47] Zhang Y. Pak C. Han Y. Ahlenius H. Zhang Z. Chanda S. (2013). Rapid single-step induction of functional neurons from human pluripotent stem cells. *Neuron* 78 785–798. 10.1016/j.neuron.2013.05.029 23764284 PMC3751803

[B48] Zhou B. Lu J. G. Siddu A. Wernig M. Sudhof T. C. (2022). Synaptogenic effect of APP-Swedish mutation in familial Alzheimer’s disease. *Sci. Transl. Med.* 14:eabn9380. 10.1126/scitranslmed.abn9380 36260691 PMC9894682

[B49] Zott B. Simon M. M. Hong W. Unger F. Chen-Engerer H. J. Frosch M. P. (2019). A vicious cycle of beta amyloid-dependent neuronal hyperactivation. *Science* 365 559–565. 10.1126/science.aay0198 31395777 PMC6690382

